# Protocol for genotyping cephalopod sex using a skin swab and quantitative PCR

**DOI:** 10.1016/j.xpro.2026.104693

**Published:** 2026-07-10

**Authors:** Frederick A. Rubino, Connor J. Gibbons, Thomas J. Mungioli, Scott T. Small, Gabrielle C. Coffing, Andrew D. Kern, Tessa G. Montague

**Affiliations:** 1 Department of Biology, Whitehead Institute, Massachusetts Institute of Technology, Cambridge, MA, USA; 2 The Mortimer B. Zuckerman Mind Brain Behavior Institute, Department of Neuroscience, Columbia University, New York, NY, USA; 3 Institute of Ecology and Evolution, University of Oregon, Eugene, OR, USA; 4 Howard Hughes Medical Institute, Columbia University, New York, NY, USA

**Keywords:** Genetics, Model Organisms, Molecular Biology

## Abstract

The coleoid cephalopods (octopus, cuttlefish, and squid) are emerging model organisms for neuroscience, development, and evolutionary biology. Determining their sex early in life is critical for population management and controlled experiments. Here, we present a protocol to non-invasively determine the sex of multiple cephalopod species as young as 3 h post-hatching using a skin swab and quantitative PCR (qPCR). We describe steps for designing qPCR primers, swabbing live animals, extracting DNA, running the qPCR, and analyzing the results.

For complete details on the use and execution of this protocol, please refer to Rubino et al.^1^

## Highlights

• Instructions for swabbing live cephalopods as early as 3 h post-hatching
• Procedures for extracting DNA from cephalopod skin swabs
• Guidance on performing qPCR-based sex determination
• Steps for designing and validating qPCR primers for new species

## Abstract (editor highlights)

Publisher’s note: Undertaking any experimental protocol requires adherence to local institutional guidelines for laboratory safety and ethics.

## Graphical abstract

**Figure:**
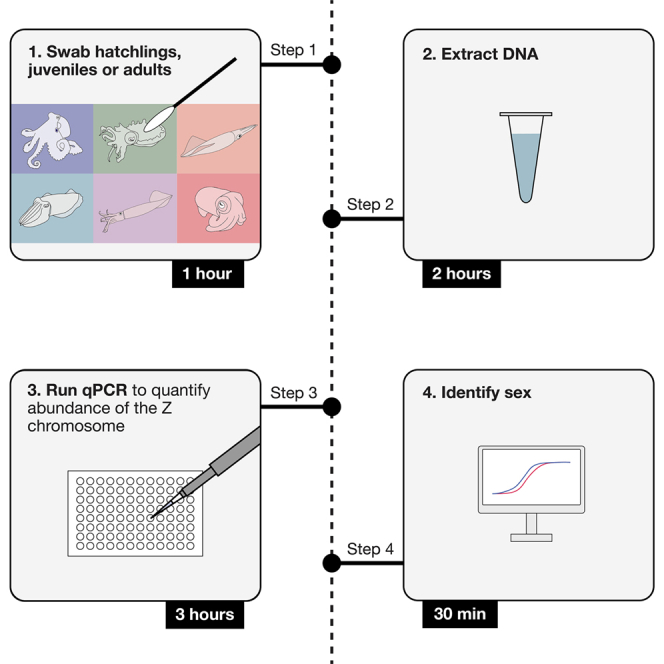

## Before you begin

Coleoid cephalopods (octopus, cuttlefish, and squid) are highly derived mollusks with the largest brain-to-body ratios among invertebrates and an array of complex behaviors, including adaptive camouflage, problem-solving, episodic-like memory, and dynamic social behaviors.^2^^,^^3^^,^^4^^,^^5^ Cephalopods and vertebrates last shared a common ancestor over 600 million years ago,^6^ yet convergently evolved large, complex brains, camera-type eyes, and closed circulatory systems.^7^ Thus, coleoid cephalopods exhibit unique biological features – including extensive RNA editing,^8^ an elaborate central and distributed nervous system,^9^ and adaptive camouflage^3^ – while providing a valuable comparative model for uncovering general principles of nervous system organization, behavior, and evolution.

Historically, cephalopod research has relied on wild-caught species such as the longfin inshore squid (*Doryteuthis pealeii*), which played a central role in the discovery of the action potential.^10^ Advances in aquaculture have enabled the culture of multiple species in the laboratory, including the California two-spot octopus (*Octopus bimaculoides*),^11^ common cuttlefish (*Sepia officinalis*),^12^ dwarf cuttlefish (*Ascarosepion bandense*),^13^ and hummingbird bobtail squid (*Euprymna berryi*),^14^ driving rapid expansion of cephalopods as experimental systems.

A key limitation in cephalopod culture is the difficulty of establishing breeding populations with defined sex ratios at early developmental stages, which can strongly influence egg production and aggression.^1^ Sex can be determined in adults by the presence of sexually dimorphic traits, such as a hectocotylus (a modified arm found in males of some cephalopod species), but these traits can be difficult or impossible to identify in juveniles. Early sex identification is therefore critical for selective breeding, including preventing unintended sperm storage, and for interpreting behavior in species that exhibit sex mimicry.^15^^,^^16^ The ability to determine sex in wild-caught fishery species, such as the northern shortfin squid (*Illex illecebrosus*) and Argentine shortfin squid (*Illex argentinus*), could also enable improved population monitoring and fisheries management.

The recent identification of a genetic sex determination system in octopus^17^ revealed a ZZ sex karyotype in males and Z0 in females. This system of sex determination can be detected via qPCR by measuring the ratio of an autosomal amplicon to a Z-linked amplicon, yielding an expected 1:0.5 ratio in females and 1:1 ratio in males.^1^ Here, we describe a non-invasive protocol for sex determination in cephalopods using skin swabs and qPCR, which can be performed on live animals as young as 3 hours post-hatching. We provide validated primer sets for *O. bimaculoides*, *S. officinalis*, *A. bandense*, *E. berryi*, *D. pealeii*, *I. illecebrosus*, and *I. argentinus*, along with guidelines for designing primers for additional species.

### Innovation

Existing methods to determine the sex of live cephalopods include examination of sex-specific anatomy (e.g. the presence of a hectocotylus) or sex-specific behaviors (e.g. zebra display^18^). However, many species lack readily observable sex-specific traits. Moreover, when present, these features typically emerge near or after sexual maturity after critical husbandry decisions have already been made, such as pairing animals before females can store sperm. Here, we describe a non-invasive approach that combines skin swabbing, previously used for genotyping in *E. berryi*,^19^ with a qPCR-based assay to quantify relative sex chromosome dosage. A conceptually similar approach has been reported in *Sepioteuthis lessoniana*^20^ but relied on post-mortem tissue. In contrast, our method uses small quantities of DNA from live animal skin swabs, enabling accurate sex determination in animals as small as 5.3 mm mantle length^1^ and across seven cephalopod species. Additionally, we provide guidelines for designing stringent qPCR primers for new species.

### Institutional permissions

Columbia University has established strict policies for the ethical use of cephalopods, including operational oversight from the Institutional Animal Care and Use Committee (IACUC). All of the cuttlefish used in this study were handled according to an approved IACUC protocol (AC-AABT8673). Experiments must be performed in accordance with local institutional and national guidelines and regulations.

### Primer design and validation (optional)

**Timing: 2 h (step 1)**

**Timing: 6–8 h (excluding primer synthesis) (step 2)**

This protocol includes validated primer sequences for seven cephalopod species: *O. bimaculoides*, *S. officinalis*, *A. bandense*, *E. berryi*, *D. pealeii*, *I. illecebrosus*, and *I. argentinus*. To adapt this protocol for additional cephalopod species, this section provides instructions for designing stringent qPCR primers and validating them. Note that some DNA sequence data are required, although a fully assembled genome is not necessary.^1^ To facilitate identification of candidate autosomal and Z-linked targets, Table S1 provides amino acid sequences for four autosomal genes and four Z-linked genes from eight cephalopod species that can be used to identify equivalent sequences in your species of interest.

**CRITICAL:** Primers must be designed to amplify genomic DNA, not cDNA or mRNA-derived sequence, because primers separated by introns may fail to amplify the intended genomic target. When only RNA-seq data are available, design multiple candidate primer pairs and screen them for amplification of a product of the expected size.

1. Primer design.
  ***Note:*** This protocol uses Geneious Prime (which uses a modified version of Primer3 2.3.7) for primer design. However, equivalent primer design software can be used with the same design criteria.
  a. Import genomic DNA sequences corresponding to candidate autosomal loci into Geneious using the “Import from File” or database retrieval functions. Each locus should be 15–30 kb in length.
    ***Note:*** Using longer sequences increases the probability of identifying primer pairs that meet the design criteria and avoid repetitive regions. Shorter loci may be sufficient when using RNA-seq–derived sequences.
    i. Include several dozen loci to increase the likelihood of identifying suitable primer pairs.
      ***Note:*** In practice, ∼24 loci are usually sufficient to give 12 good primer pair candidates, but it is easier to include more loci and design an excess of primer pairs at this step.
    ii. When possible, select loci distributed across the chromosome to minimize regional bias.
  b. Use the Primer Design tool to generate a candidate qPCR primer pair against each locus with the following parameters:

**Table:** 

Parameter	Setting
Product size	100 - 200 bp
Optimal primer length	20 bp (18 - 27 bp)
Optimal melting temperature (Tm)	59°C (57 - 63°C)
Optimal GC content	50% (40 - 60%)
GC clamp	1
Max poly-X	3
Max dimer Tm	30°C
    i. Set primer pair penalty weights high (e.g. 5) for individual and pair “dimer Tm” parameters under “Primer Picking Weights” in Geneious Prime to minimize primer–dimer formation.
  c. Select primer pairs that lack predicted secondary structure, primer-dimer formation, and significant off-target binding within the available sequence dataset.
    i. Screen candidate primer pairs using OligoAnalyzer Tool for hairpins, self-dimers, and hetero-dimers.
    ii. Reject primer pairs with predicted secondary structures with a Tm >30°C or with ≥4 consecutive complementary bases in primer–dimers, particularly at the 3′ end.
    iii. Perform a BLAST search of each predicted amplicon against the reference genome (or available sequencing data) to assess off-target binding.
    iv. Reject primers that bind repetitive regions and could generate similarly sized off-target products.
  d. Repeat steps a-c for candidate sex-linked (Z-linked) loci.
  e. Design a minimum of 12 primer pairs for the autosomal loci and 12 primer pairs for the Z-linked loci (24 total primer pairs recommended).
    ***Note:*** Screening primer pairs in groups of 8 or 12 simplifies plate setup and ordering while increasing the likelihood of identifying suitable primer combinations.
2. Primer validation.
  ***Note:*** Candidate primer pairs are screened to identify those with high amplification efficiency and specificity that produce clear dosage differences between sexes.
  a. Obtain genomic DNA (gDNA) from confirmed male and female individuals of the target species for validation.
    ***Note:*** For instance, extract gDNA from post-mortem tissue (e.g. arm tips) of phenotypically sexed animals by following the manufacturer’s instructions of the Monarch Spin gDNA Extraction Kit. This should yield sufficient DNA for multiple rounds of primer testing.
  b. Make a 20× stock primer mix in nuclease-free water – 5 μM forward & 5 μM reverse primer.
  c. Set up qPCR reactions for each primer pair using gDNA from one confirmed male and one confirmed female individual.
    ***Note:*** Include a no-template control (NTC) for each primer pair (see **“**qPCR amplification of autosome and sex chromosome loci**”** for complete details for qPCR reactions).
    For each reaction:
    i. Use 2 μL of 1 ng/μL gDNA as template (in a 7 μL reaction).
    ii. Use primers at a final concentration of 0.25 μM.
    iii. Perform 4 technical replicates per condition and calculate the mean Cq value.
  d. Evaluate amplification curves and discard primer pairs that show:
    i. No amplification.
    ii. Late amplification (e.g. Cq > 35).
    iii. Amplification in NTCs before Cq = 30.
    iv. Early amplification.
      ***Note:*** Later amplification in NTCs, or no amplification at all, is preferred, as any NTC amplification will limit the range of gDNA concentrations you will be able to test.
      ***Note:*** Most primer pairs should give Cq values that cluster within a small range (±2 Cq from the median, see Figure S1 for an example). Early Cq values likely indicate off-target binding, primer-dimer amplification, or amplifying a highly duplicated region.
  e. Examine melt curves and discard primer pairs that produce multiple peaks or evidence of non-specific amplification (see Figure S2 for an example).
    i. Accept only primer pairs with single, sharp peaks (Figure S2) that are consistent between male and female samples and absent in NTCs.
  f. Resolve PCR products on a 2% agarose gel to confirm a single amplicon of the expected size using a 100 bp ladder.
  g. Confirm amplicon identity by sequencing.
    ***Note:*** You can use Sanger sequencing (forward and reverse reactions) or a direct sequencing approach (e.g. nanopore “Premium PCR Sequencing” service by Plasmidsaurus).
  h. For primer pairs that pass validation (steps c-g), compare Cq values between male and female samples:
    i. For each possible autosome/sex primer combination, compute the ΔCq value as:
      Cq(auto)−Cq(sex)
      for both male and female samples.
      ***Note:*** Males have two copies of the Z chromosome (ZZ), whereas females have one (Z0). A two-fold difference in template corresponds to a ∼1 cycle difference in Cq.
    ii. Identify combinations of autosomal and sex-linked primer pairs that satisfy ΔΔCq ≈ +1:
      ΔCq(Auto−Sex,male)−ΔCq(Auto−Sex,female)≈+1.0
    iii. Accept combinations of primer pairs with ΔΔCq values between 0.8 and 1.2 for further analysis.
    iv. Select ≥4 primer pairs per chromosome type that meet these criteria.
      ***Note:*** Individual primer pairs are expected to perform well in multiple autosome–sex primer combinations.
  i. Determine amplification efficiency for all primer pairs that passed the validation and selection criteria above.
    ***Note:*** This will inform what concentration ranges the assay will work within.
    i. Prepare a gDNA standard curve from 10 ng/μL to 0.001 ng/μL in an 8-well strip using 3.126× (√(10)×) serial dilutions in nuclease-free water.
    ii. Run qPCR reactions as described above (step 2c) and record Cq values for each template concentration.
    iii. Plot the standard curve as Cq versus log10(template concentration).
      ***Note:*** Standard curves should be linear over approximately Cq 20–30.
    iv. Identify the linear range for each primer pair by fitting a linear regression:
      Cq=m×log10([template])+b
      ***Note:*** The assay will make reliable predictions only within this Cq value range for this primer pair.
    v. Calculate primer efficiency:
      $$\mathrm{Efficiency}\;(\%)=(10^{-1/m}-1)\;\times 100$$
      ***Note:*** 100% efficiency corresponds to slope = −3.32. Acceptable range: 90–110% efficiency (slopes −3.6 to −3.1).
    vi. Discard primer pairs with efficiencies outside this range and retain those with the highest performance.
  j. Using the best-performing primer pairs, perform sex determination on 8 animals (ideally 4 female and 4 male) using gDNA normalized to 1 ng/μL:
    i. See section “qPCR amplification of autosome and sex chromosome loci” for complete details on qPCR reactions and analysis.
    ii. Randomize gDNA samples so that sample identity (sex) is blinded during qPCR setup and analysis.
    iii. Follow sections “qPCR amplification of autosome and sex chromosome loci” and “qPCR analysis” to obtain ΔCq(A–S) for all 8 animals, for all primer pair combinations.
    iv. Ensure that Cq values fall within the validated linear range for each primer pair (step 2i-iv).
      ***Note:*** If they don’t, adjust the template concentrations by dilution or concentration (e.g. a two-fold dilution will increase the Cq value by ∼1).
    v. For each primer pair combination (autosome & sex chromosome) assign sex to all 8 samples based on ΔCq values, following the steps in section “qPCR analysis”.
  k. Identify primer pair combinations that correctly classify all samples (100% accuracy); these are validated for future genotyping.
    ***Note:*** Maintain a record of genotyping performance for all future trials and note any failures for each primer pair combination.
    ***Note:*** Depending on the error tolerance of your application, you may want to perform more extensive trials at this point or include primers to target multiple autosomal and sex loci in your assay.
    ***Note:*** If no primer pairs pass validation, redesign additional candidates or consider alternative loci.

### Build a housing tray for temporary individual isolation

**Timing: ∼6 h**

To maintain the identity of individual cephalopods after skin swabbing and before sex assignment results, construct a compact housing tray that enables temporary (24-72 h) high-density isolation of up to 20 cephalopods within a single 20″ × 15″ footprint. Because juvenile and subadult cephalopods can pass through extremely small openings, construct the housing system with tight tolerances and secure plumbing.

**Figure 1:**
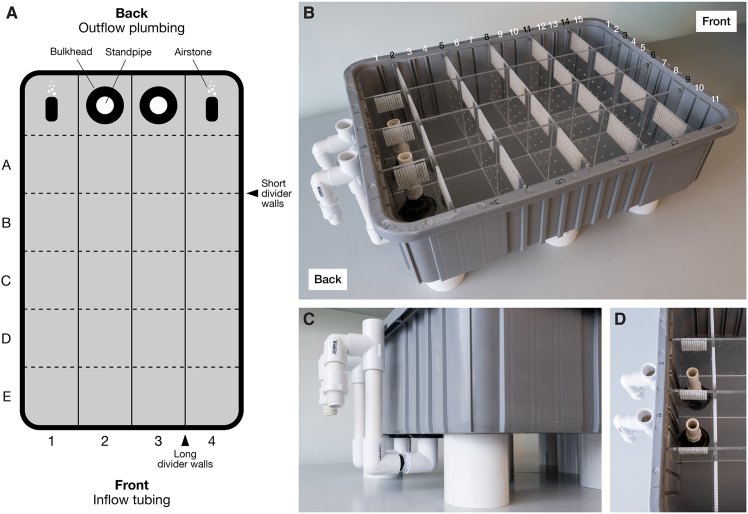
High-density housing tray for temporary isolation of animals (A) Schematic of housing tray containing 20 usable compartments for individual animals. The footprint is compatible with most aquatic rack systems. (B) Assembled housing tray. Interlocking ¼” cast acrylic dividers fit tightly into molded slots to prevent animal movement between compartments. (C) External bulkhead and PVC standpipe assembly used to establish controlled overflow and consistent water level. (D) Internal view of bulkhead placement and vertical standpipes that set the operating water level.
    ii. 5 short divider walls (spanning left to right between long dividers, Figure 1A).
    iii. Use laser cutting settings optimized for ¼″ cast acrylic, adjusting power, speed, and passes according to the specific laser cutter being used.
  b. Ensure slot tolerances are tight enough to prevent lateral movement once inserted into the divider box (tray).
    i. To test fit: insert a divider fully into the molded slots of the empty tray (see Figure 1B for slot locations) and attempt to gently shift the divider side-to-side.
    ii. Fit is acceptable if the divider remains upright with minimal lateral movement and the divider does not shift when the tray is tapped.
    iii. If dividers are too loose, re-cut with slightly increased length (+0.1–0.2 mm).

3. Laser-cut divider walls from ¼” cast acrylic.
  a. Use the provided SVG file (Data S1) to cut:
    i. 3 long divider walls (spanning the full length of the tray, front to back, Figure 1A).

**CRITICAL:** Dividers must fit snugly into the tray’s molded slots. Even small gaps can permit hatchlings or juveniles to squeeze between compartments.

***Note:*** If a laser cutter is not available, acrylic components can be custom ordered from commercial laser cutting services.

4. Prepare outflow plumbing with bulkhead installation.
  a. Identify the back of the tray as the side that will be closest to the sump or plumbed structure that will send the outflowing system water directly to the sump for filtration (Figure 1A).
  b. Using a handheld electric drill with a hole saw bit, drill two ⅞” holes in the bottom of the back-most center compartments (Figure 1A).
    i. Center each hole within its compartment floor.
    ii. Maintain at least 0.5″ clearance from divider walls to ensure proper bulkhead sealing (Figure 1D).
  c. Install the threaded bulkhead such that the flush flange sits on the exterior of the tray and the threaded portion protrudes into the interior (Figure 1).
    i. Place the gasket on the water-facing (internal) side.

***Note:*** The bulkheads are installed in a non-standard orientation due to space constraints of the existing aquatic system. Reversing the orientation of the threaded side is acceptable, and may be preferred if space permits, provided the gasket remains seated on the water-facing side.

5. Install outflow plumbing.
  a. Apply Teflon tape to all threaded components before installation:
    i. Wrap clockwise (in the direction of tightening).
    ii. Use at least one overlapping layer.
  b. Attach a ½″ PVC male threaded adapter to each bulkhead from inside the tray (Figure 1A).
  c. Cut and insert the PVC pipe into the male adapters to set the operating water level.
    ***Note:*** The height of the internal standpipe determines the tray water level. A PVC length of 3 ¾” will create a water depth of approximately 5”.
  d. Attach a 90^o^ threaded elbow fitting to each bulkhead on the external side using a ½″ threaded nipple (Figure 1C):
    i. Orient elbows to direct flow toward the external drain or outflow line returning to the sump.
    ii. If utilizing a conventional aquatic rack system where the outflow plumbing sits at or above the bulkhead height, account for the vertical difference by continuing to step 5e.
    iii. If the outflow plumbing sits below the bulkhead height, plumb a direct downwards path and proceed to step 6.
  e. Create a U-shaped section of plumbing utilizing a 90^o^ elbow, and straight piece of ½″ PVC to reach the required height of your specific aquatic rack system (Figure 1C).
    i. Connect the 90^o^ threaded elbows utilizing ½″ threaded nipples.
  f. Create a hook utilizing a ½″ tee fitting and slip 90^o^ elbow to direct water outflow into the overflow compartment of the aquatic rack system (Figure 1C).
    i. The tee fitting is utilized to break any siphon and prevent issues with air locking in the outflow plumbing.
      ***Note:*** The installation of a “U” and “hook” structure in the outflow plumbing is required to accommodate constraints of the existing aquatic system and maintain proper water outflow to the sump. Where possible, a simpler configuration – such as attaching a ½” 90° PVC barb to the bulkhead and routing aquarium-safe tubing directly to the sump or filtration area – may be more user-friendly.
6. Insert divider walls.
  a. Place long dividers into vertical slots 3, 6, and 9 (left to right, Figure 1B).
  b. Place short dividers into horizontal slots 2, 5, 8, 11, and 14 (front to back, Figure 1B).

***Note:*** The completed layout creates 20 isolated compartments for animals plus 4 compartments for the outflow and air.

7. Stabilize and level the tray.
  a. Elevate the tray using PVC couplings as risers beneath the base.
    ***Note:*** Risers are placed in consistent intervals along the outside perimeter and middle of the tray to prevent the bottom of the tray from bowing.
    ***Note:*** This elevation provides clearance for outflow plumbing and ensures compatibility with aquatic rack systems that use elevated outflow troughs.
    i. Ensure the tray is level by placing a bubble level across both the front–back and left–right axes of the tray.
      ***Note:*** Ensuring the tray is level prevents uneven water heights between compartments, which can affect water exchange and animal conditions. If a bubble level isn’t available, visually compare water height across compartments after partial filling.
    ii. If the tray is not level, adjust by repositioning risers or adding shims beneath the low corners until the water height is uniform across all compartments.
8. Install aeration.
  a. Place airstones in rear corner compartments (Figure 1A) to promote water mixing and oxygenation.
9. Test the system before introducing animals:
  a. Fill the tray with system water and allow it to run for at least 30 min.
  b. Check all bulkheads and threaded connections for leaks.
  c. Confirm equal water height across compartments.
    i. If water levels are unequal, verify that the tray is level, that standpipes are of equal height and fully seated, and that no obstructions are present in the outflow.
  d. Confirm the bottom of the tray has not bowed and there are no gaps at the bottom of the acrylic of each compartment.
  e. Measure temperature, salinity, pH, and dissolved oxygen to ensure consistency with home tanks.

**CRITICAL:** Confirm that there are still no gaps between the dividers, tray walls, and tray bottom now that the tray is bearing weight.

***Note:*** Thread sealant tape and properly seated gaskets are typically sufficient to ensure watertight seals. If leaks are observed, drain the tray and apply a small amount of aquarium-safe silicone to the leaking interface and allow it to cure fully (≥48 h) before refilling.

10. Assign each compartment a grid coordinate (e.g. A1, B1) for ease of identification (Figure 1A).
  a. Transfer animals immediately after swabbing to their assigned compartment.
  b. Maintain animals in the tray only until genotyping results are available and new housing assignments are determined.

## Key resources table

**Table:** 

REAGENT or RESOURCE	SOURCE	IDENTIFIER
**Chemicals, peptides, and recombinant proteins**		

APExBIO 2× SYBR Green qPCR Master Mix	APExBIO	K1070
Agarose	Sigma-Aldrich	A7174-10G
50× Tris-Acetate-EDTA (TAE) solution	Thermo Scientific	B49
SYBR Safe DNA gel stain	Fisher Scientific	S33102
100 bp DNA ladder	New England Biolabs	N3231S
Nuclease-free water	Ambion	AM9930

**Critical commercial assays**		

Monarch Spin gDNA Extraction Kit	New England Biolabs	T3010L

**Experimental models: Organisms/strains**		

*Ascarosepion bandense*	Columbia University	N/A

**Oligonucleotides**		

See Table 1.	–	–

**Software and algorithms**		

Geneious Prime 2025.2.2	N/A	https://www.geneious.com
BLAST	N/A	https://blast.ncbi.nlm.nih.gov/Blast.cgi
QuantStudio Design & Analysis Software v2.2.0	Applied Biosystems	N/A
IDT OligoAnalyzer	IDT	https://www.idtdna.com/calc/analyzer

**Other**		

20V Compact Drill	Dewalt	885911723633
⅞″ Hole Saw Bit	Grainger	3ERF4
Divider Box - 20″ × 15″ × 6″	ULINE	S-19496BLU
½″ FPT × FPT Bulkhead. Quantity: 2	Lifegard Aquatics	R270900
½″ Slip × ½″ Male NPT Adapter. Quantity: 2	Grainger	22FJ19
½″ × 10 PVC Schedule 40 Pipe NOTE: there will be leftover PVC after construction	Grainger	5AFJ2
½″ FPT × ½″ MPT 90^o^ Elbow. Quantity: 2	Grainger	60UD05
½″ FPT × ½″ Slip 90^o^ Elbow. Quantity: 2	Grainger	22FJ88
Pipe Thread Sealant Tape (Teflon Tape)	Grainger	3PDL5
¼″ Clear Cast Acrylic Sheet (for laser cutting) - 32″ × 18″. Quantity: 2	TAP Plastics	N/A
1½″ PVC Coupling (as risers). Quantity: 5	Grainger	22FM12
½″ Slip × ½″ Slip x ½″ Slip Tee Pipe Fitting. Quantity: 2	Grainger	22FL02
½″ Slip × ½″ Slip 90^o^ Elbow. Quantity: 2	Grainger	22FJ90
½″ Threaded Pipe Nipple. Quantity: 4	Grainger	3TNV7
Dow 795 Silicone Building Sealant - 10.3 Fluid Ounce Cartridge	PK Supplies	795BL
Airstones	Bulk Reef Supply	256276
1.5 mL Safe-Lock tubes	Eppendorf	0030123611
6″ Sterile Standard Foam Swab w/Polystyrene Handle	Puritan	25-1506 1PF 100
Delicate Species Fish Net, 3″ Fine Mesh	Seachem	67103228
Polypropylene Measuring Jug, 5000 mL	Eisco	S23802
Microfiber Towel, 11.5″ × 11.5″, 12 Pack	S&T Inc	075182099418
SnapStrip 8-Strip Standard PCR Tubes with Individually Attached Flat Caps	GeneMate	490003-706
Nanodrop or equivalent	Thermofisher Scientific	ND-ONE-W
ThermoMixer or equivalent or a heat block + vortex	Eppendorf	2231001127
Mini Dry Bath or equivalent	Fisherbrand	14-955-221
Mini Dry Bath 1.5 mL Accessory Block	Fisherbrand	14-955-224
MicroAmp EnduraPlate Optical 384-Well Clear Reaction Plates or equivalent	Applied Biosystems	4483285
MicroAmp Optical Adhesive Film or equivalent	Applied Biosystems	4311971
QuantStudio 384-well qPCR machine or equivalent	Applied Biosystems	A28140
Sartorius Picus 2 Electronic Multichannel Pipette (120 μL) or equivalent	Sartorius	LH-747421
Sartorius Picus 2 Electronic Multichannel Pipette (10 μL) or equivalent	Sartorius	LH-747441
Benchmark PLATEFUGE or equivalent	Benchmark Scientific	C2000
4″ Deluxe Soft Rubber Brayer	Speedball	4126
Wide Mini-Sub Cell GT Cell DNA electrophoresis system or equivalent	Bio-Rad	1704405

**Table 1:** qPCR primer sequences for cuttlefish, octopus and squid species

Organism	Primer name	Primer sequence (5’→3’)
***Ascarosepionbandense***	Abandense_qPCR_auto_F	GGTGTTGTTCGCTCAGTTATC
	Abandense_qPCR_auto_R	GTGTTCATGTCGCCATCTTATC
	Abandense_qPCR_sex_F	GGCCGTCCTCTACTTGTAATG
	Abandense_qPCR_sex_R	AGTAGCTGTGTGGTTGAGAAG
***Octopus bimaculoides***	Obimaculoides_qPCR_auto_F	TTGTTTGGACCTTGGGCTTATAG
	Obimaculoides_qPCR_auto_R	CTGTCATGAACCCTGGTGTATTC
	Obimaculoides_qPCR_sex_F	CCTCACCACTGGATGCAATTAAG
	Obimaculoides_qPCR_sex_R	GCCAATCCGTCCAACCTATAC
***Sepia officinalis***	Sofficinalis_qPCR_auto_F	TTTGCCACTGTGTCCCTTTATAC
	Sofficinalis_qPCR_auto_R	ACACACACAGGCTGCTTATTG
	Sofficinalis_qPCR_sex_F	TTTCAACCCATCTGCGTCTATAG
	Sofficinalis_qPCR_sex_R	ACTCCTCTCGTTGCATGATTAC
***Euprymna berryi***	Eberryi_qPCR_auto_F	CTTTCGCCACGCCTGATATAC
	Eberryi_qPCR_auto_R	CAGCAGCTTCTTTCCCAGATAAG
	Eberryi_qPCR_sex_F	CTGCCCAGCGAATTGTTTATTG
	Eberryi_qPCR_sex_R	TCCGGCGTCTAGGGATTTAG
***Doryteuthis pealeii***	Dpealeii_qPCR_auto_F	CACTTCAGCCCGATGGAATAAG
	Dpealeii_qPCR_auto_R	CTTTGTAAATGCCGCACCTATATC
	Dpealeii_qPCR_sex_F	GGAGTCTGAGGTCCGAGATATAG
	Dpealeii_qPCR_sex_R	GCCGAGACCACAAACAATAAC
***Illex illecebrosus & Illex argentinus***	Illex_qPCR_auto_F	AAAACTCCCGACGTCTTGAA
	Illex_qPCR_auto_R	GGCCATCCTGGTAGACAAGA
	Illex_qPCR_sex_F	AATCACCCCAACCAGATGAA
	Illex_qPCR_sex_R	CTCCTGGACCTGGAATGAAA

## Step-by-step method details

### Prepare tissue lysis buffer

**Timing: 15 min**

1. Label a series of 1.5 mL Safe-Lock tubes for the total number of animals being genotyped.

***Note:*** Safe-Lock tubes are recommended as samples are shaken at high speed during incubation, which can cause standard tube lids to open.

2. Calculate a master mix for the total number of samples (+10% for pipetting error):

**Table:** 

Reagent	Per reaction
Tissue lysis buffer (from *Monarch Spin gDNA Extraction Kit*)	200 μL
Proteinase K (20 mg/mL) (from *Monarch Spin gDNA Extraction Kit*)	3 μL

***Note:*** For DNA extractions from a piece of tissue (e.g. an arm tip) instead of a skin swab, use 10 μL of Proteinase K instead of 3 μL.

3. Mix thoroughly by gentle pipetting or inversion.
4. Aliquot 200 μL of the master mix into each labeled tube and proceed directly to the next step.

### Skin swabbing

**Timing: 1 h (for 24 samples)**

In this step, skin cells are collected from live, unanesthetized cephalopods for DNA extraction. This step is best performed with two people: one to handle the animal and one to perform the swabbing.

5. Open a sterile foam swab, dip it in tank water to pre-soak it, then shake off the excess water.

***Note:*** A dry swab can damage the skin of small cephalopods.

**Figure 2:**
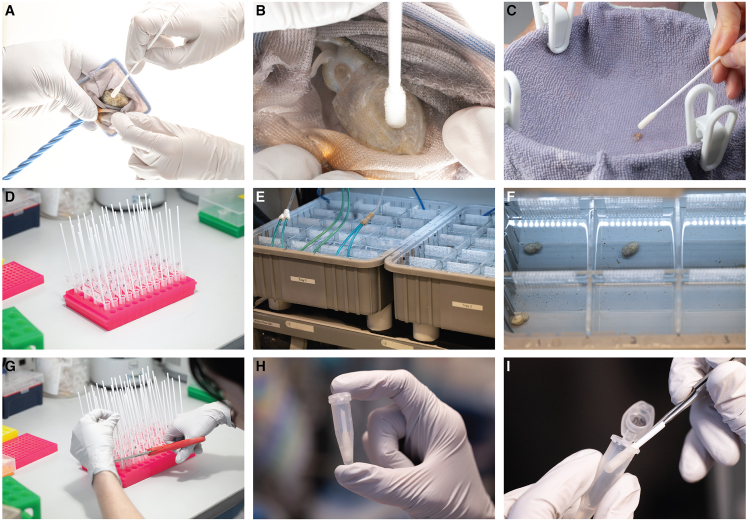
Skin swabbing and DNA extraction (A) Juvenile or adult cephalopods are caught in a net and gently swabbed on the dorsal mantle using a pre-soaked swab. (B) The animal is rotated and the ventral surface is swabbed with slightly greater pressure. (C) For hatchlings, animals are transferred onto a microfiber towel suspended in a jug of seawater such that the gills remain submerged. The dorsal and ventral surfaces are swabbed using extremely light pressure. (D) Each swab is placed into a tube containing pre-mixed Tissue Lysis Buffer and Proteinase K. (E) Immediately after swabbing, each animal is transferred to a housing tray (see “build a housing tray for temporary individual isolation”). (F) Animals are maintained in isolation to preserve identity. (G) The swab shaft is cut using scissors. (H) Shortening the swab shaft allows the tube lid to close. (I) After incubation with shaking for 1 hour, the swab is pressed against the side of the tube to recover residual liquid.
    Methods video S1. Swabbing a live juvenile cuttlefish, related to “Skin swabbing” step 6b
  c. Gently rotate the animal so its ventral side is facing upwards.
  d. Rotate the swab 180° to use a clean side of the tip. Gently swab the ventral skin in a series of parallel strokes to sample from the entire ventral surface (Figure 2B).
    ***Note:*** Slightly greater pressure can be applied compared to the dorsal surface.
7. For swabbing hatchlings:
  a. Place the hatchling on a microfiber towel suspended ∼5 mm below the surface of artificial seawater in a 5 L jug (Figure 2C).
  b. Gently lift the towel edges to expose the mantle above water.
  c. Using a pre-soaked sterile foam swab, lightly swab the dorsal mantle in a small series of parallel strokes to sample from the entire mantle surface.
    **CRITICAL:** Use extremely light pressure to avoid damaging the skin.
    **CRITICAL:** Carefully avoid passing the swab over the same region more than once.
  d. Rotate the animal and repeat on the ventral side with the other side of the swab.
8. Place the swab face down in the corresponding Safe-Lock tube containing the lysis buffer/proteinase K mix and leave it submerged until you’ve completed all swabbing (Figure 2D).
9. Return the animal to its designated tank or position in the housing tray (Figures 2E and 2F).

***Note:*** Recently swabbed skin may appear discolored or show transient markings. This is normal and typically resolves within minutes. Bright white patches indicate skin damage; in such cases, healing occurs within 24–48 h, but reduced pressure should be used for subsequent animals.

6. For swabbing juveniles and adults:
  a. Gently catch the animal using a soft net and hold it within the net (out of water), with its dorsal mantle facing up.
  b. Using one side of the swab tip, gently swab the dorsal skin in a series of parallel strokes to sample the entire mantle surface. Avoid passing the swab over the same area more than once (Figure 2A and Methods video S1).
    **CRITICAL:** The dorsal skin is very delicate in young animals (<2 months old), so be extremely gentle. Avoid swabbing the skin all the way to the anterior end of the mantle, as this is the most delicate region of the skin.
    ***Note:*** Temporary skin discoloration immediately after swabbing is normal (Methods video S1). However, bright white patches indicate skin damage that has exposed the underlying cuttlebone and should be avoided by using gentler swabbing pressure.
    ***Note:*** In adults (>4 months), a mucus layer is present, and additional pressure is required to collect sufficient cells.

10. Repeat steps 5–9 for each animal.

### DNA extraction

**Timing: 2 h**

DNA is extracted from skin swabs collected in tissue lysis buffer.

11. Transfer the rack of Safe-Lock tubes (with foam swabs inside) to the bench.
12. For each tube, lift the swab a few millimeters and cut the shaft using clean scissors so that the tube lid can be fully closed (Figures 2G and 2H).
13. Incubate the tubes in a thermomixer at 56°C with shaking at 1,400 rpm for 1 hour.

***Note:*** If a thermomixer is not available, incubate tubes in a 56°C heat block and vortex vigorously every 5–10 min to facilitate lysis.

14. After incubation, briefly centrifuge the tubes to collect condensation and liquid at the bottom.
15. Using a gloved hand or forceps, grip the swab securely and press and drag it against the side of the tube to extract as much liquid as possible (Figure 2I).
16. Discard the swab and repeat for all samples.
17. Proceed with genomic DNA purification using the Monarch Spin gDNA Extraction Kit:
  ***Note:*** You can skip the RNase A step.
  a. Add 400 μL of gDNA Binding Buffer to each tube and mix thoroughly by pulse-vortexing for 5–10 sec.
  b. Pour the lysate/binding buffer mix (∼600 μL) into a gDNA Purification Column pre-inserted into a collection tube.
  c. Centrifuge: first for 3 min at 1,000 × *g* to bind gDNA (no need to empty the collection tubes or remove from centrifuge) and then for 1 minute at maximum speed (>12,000 × *g*) to clear the membrane. Discard the flow-through.
  d. Add 500 μL gDNA Wash Buffer, centrifuge immediately for 1 minute at maximum speed (12,000 × *g*), and discard the flow-through.
  e. Reinsert the column into the collection tube, add 500 μL gDNA Wash Buffer and close the cap.
  f. Centrifuge immediately for 1 minute at maximum speed (>12,000 × *g*), then discard the collection tube and flow-through.
  g. Place the gDNA Purification Column in a DNase-free 1.5 mL microfuge tube.
  h. Add preheated (60°C) gDNA Elution Buffer directly to the center of the column membrane using the following volumes:

**Table:** 

Sample type	Elution volume
Hatchling	50 μL
Juvenile	200 μL
Adult	50 μL
  i. Close the cap and incubate at room temperature for at least 5 minutes.
  j. Centrifuge for 1 minute at maximum speed (>12,000 × *g*) to elute the gDNA.
    ***Note:*** Typical yields from animals with a mantle length of 10-20 mm range from approximately 225 ng-1.1 μg total DNA. However, when eluted in 200 μL, the DNA concentration is usually below the accurate detection threshold of a Nanodrop.
    **CRITICAL:** There is no need to normalize the DNA concentrations.
18. For ease of multichannel pipetting at the next step, transfer samples into 8-strip PCR tubes (e.g. GeneMate SnapStrip 8-Strip Standard PCR Tubes with Individually Attached Flat Caps).

**Pause point:** DNA samples can be stored at −20°C if not used immediately.

### qPCR amplification of autosome and sex chromosome loci

**Timing: 3 h**

Extracted genomic DNA is amplified via qPCR using primers targeting an autosomal locus, “A”, and a sex chromosomal locus, “S”, to obtain quantification cycle (Cq) values. Validated primer sets are provided in Table 1.

19. Prepare 20× primer mixes for autosomal (“A”) and sex chromosomal (“S”) loci by combining forward and reverse primers in nuclease-free water to a final concentration of 5 μM each. Keep on ice.

***Note:*** Primer mixes can be stored at −20°C and reused for several months.

20. Thaw qPCR reagents on ice.
21. Prepare two master mixes, “A” & “S”. For N animals, prepare enough of **each** master mix (“A” & “S”) for 4×N reactions + 10% excess to account for pipetting loss.

**Table:** 

Reagent	Per reaction	Final
2× APExBIO SYBR Green qPCR mix (2×)	3.5 μL	1×
50× ROX reference dye (*from APExBIO SYBR Green qPCR mix*)	0.14 μL	1×
0.35 μL primer mix “A” or “S” (5 μM fwd, 5 μM rev)	0.35 μL	0.25 μM fwd primer 0.25 μM rev primer
Nuclease-free water	1.01 μL	–
**Total**	5 μL	–

***Note:*** Reactions will have a final volume of 7 μL: 5 μL master mix + 2 μL sample DNA.

***Note:*** For the most reliable qPCR results, work quickly and prepare master mixes immediately before adding sample DNA and running qPCR amplification. Minimize the time at room temperature by keeping reagents (and, optionally, the 384-well plate) on ice.

22. Using a multichannel pipette, load 5 μL master mix into wells of the 384-well plate. For each animal, perform 4 technical replicates per primer set.

**Figure 3:**
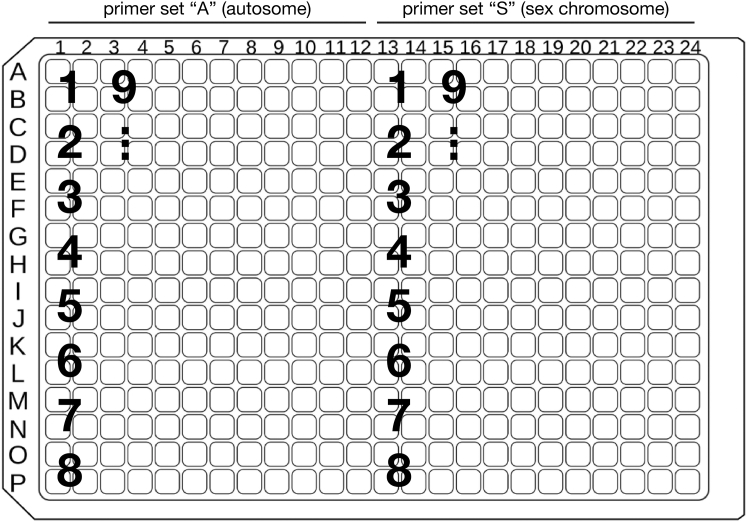
Recommended sample arrangement of a 384-well qPCR plate The locations of samples for animals 1–9 are indicated by large black letters. Four technical replicates are performed per primer set for each animal.

***Note:*** A 384-well plate layout can accommodate up to 48 animals (Figure 3).

***Note:*** An electronic repeating multichannel pipette is recommended to improve speed and dispensing accuracy.

23. Centrifuge the plate at 400 × *g* for 1 minute to collect the master mix at the bottom of the wells.

***Note:*** If an optically clear plate is used, visibly confirm the volume in each well is identical and no bubbles are trapped in the bottom of the well. Bubbles will interfere with the fluorescence readings of the qPCR machine.

24. Add 2 μL DNA sample to each well of the 384-well plate according to the schematic (Figure 3).

***Note:*** Load the sample as a 2 μL bead along the sidewall of the tube using a repeating electronic multichannel pipette for the most consistent results. Continuously monitor the volume inside each pipette tip to ensure accurate dispensing. Examine pipette tips after each dispensed volume to ensure that sample fully entered the well and does not remain as a bead on the pipette tip.

***Note:*** Extra DNA samples can be stored at −20 °C for future use.

25. Gently tap the plate to bring droplets to the bottom. Seal with optical adhesive film, first by hand and then using a roller to ensure a tight seal.
26. Centrifuge the plate at 400 × *g* briefly to collect the volume at the bottom of the wells.
27. To mix, hold the plate and use single swift motions to shake the contents toward the lid, and toward the bottom of the wells, alternately. Repeat 5 times.

***Note:*** Avoid vortexing the plate as this can introduce bubbles and compromise the activity of the polymerase.

28. Centrifuge the plate at 400 × *g* for 1 min to collect the master mix at the bottom of the wells.

***Note:*** Confirm again that wells are free of bubbles and volumes are consistent.

**Figure 4:**
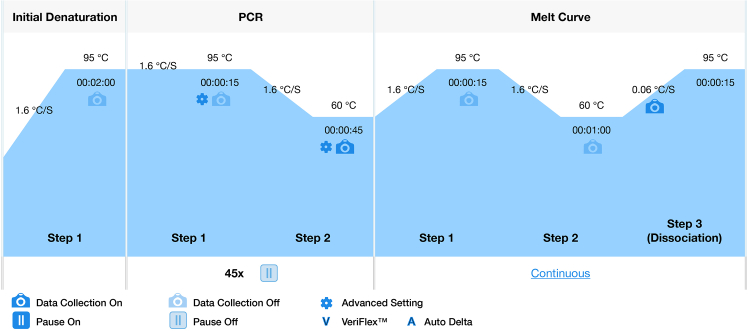
PCR protocol from QuantStudio 5 Design and Analysis software v2.2.0

***Note:*** The following is for APExBIO SYBR green polymerase on QuantStudio 5, but follow your instrument and polymerasedocumentation if they differ:

29. Perform qPCR using an appropriate instrument with the following cycling conditions (Figure 4).

**Table:** 

Steps	Temperature	Time	Cycles
Initial denaturation	95°C	2 min	1
PCR	95°C	15 s	45
	60°C	45 s	
Melt curve	95°C	15s	1
	60°C	1 min	
	95°C (at a rate of 0.06°C/s)	15s	

Set heated lid to 105°C and ramp rate to 1.6°C/s unless otherwise specified. ROX should be selected as the passive reference dye.

***Note:*** Regardless of manufacturer or model, ensure the instrument will calculate Cq values and record melt curve data.

### qPCR analysis

**Timing: 30 min**

Analyze the qPCR results to determine which animals are males and females.

**Figure 5:**
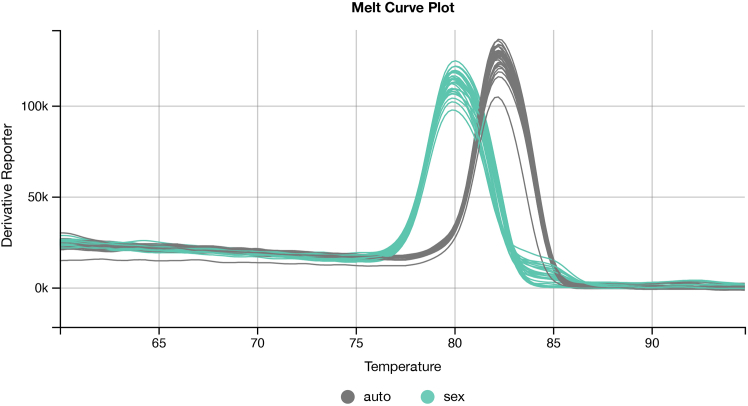
Melt curve plots of amplicons from autosome- and sex chromosome-targeting primers against 8 individuals

***Note:*** For newly designed primers, confirm specificity by resolving amplicons on a 2% agarose gel and/or sequencing products (see “primer design” and “primer validation” sections).

***Note:*** Reasons for incorrect melting profile include low sample DNA concentration, or an off-target amplification. In the latter case, consider redesigning primers. Refer to “primer design” and “primer validation” sections.

**Figure 6:**
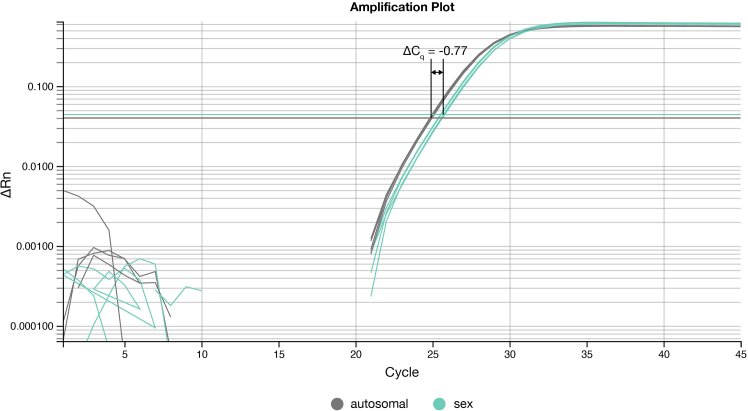
qPCR amplification plots from four technical replicates using autosomal (dark grey) and sex-linked (teal) primers for a single individual Horizontal dark grey and teal lines indicate fluorescence thresholds, and black vertical lines mark the corresponding Cq values. ΔCq is defined as the difference between autosomal and sex-linked Cq values.

***Note:*** Remove up to one outlier within a replicate set (e.g. Cq deviating by >1 cycle), which typically reflects a pipetting error (see Figure S3 for an example).

***Note:*** Aberrant amplification curves may result from improperly loaded wells (e.g. bubbles), or from impure DNA preparation. For the latter, dilute the sample DNA 10-fold and repeat the amplification or prepare a new DNA sample.

***Note:*** Ensure all Cq values fall within the linear range of the primer pairs used. Otherwise, adjust template concentration accordingly. Refer to the “primer validation” section.

***Note:*** For best results when genotyping multiple samples, use gDNA of roughly equal concentration. Preparing samples as suggested in the section “DNA extraction” should give reasonably similar concentrations for all sample types.

30. Inspect the melt curves to ensure a single melting point is observed for all samples of a given primer set (Figure 5). Discard any replicates with aberrant melt curves from the analysis.

31. Inspect the amplification plots of all samples to ensure a single slope giving a reliable Cq determination (Figure 6). Discard any replicates with aberrant amplification curves from the analysis.

32. For each animal, calculate from the four replicates the mean Cq value (A_Cq and S_Cq) and the standard error of the Cq value (A_SE and S_SE) for each primer pair.

***Note:*** For *A. bandense*, following this protocol should produce Cq values in the range of 22-25 and standard error values below 0.1. The difference between a ZZ and Z0 individual is expected to be only 1 cycle; therefore, if standard error values rise above 0.25 cycles, the ability to discern males and females will diminish.

**Figure 7:**
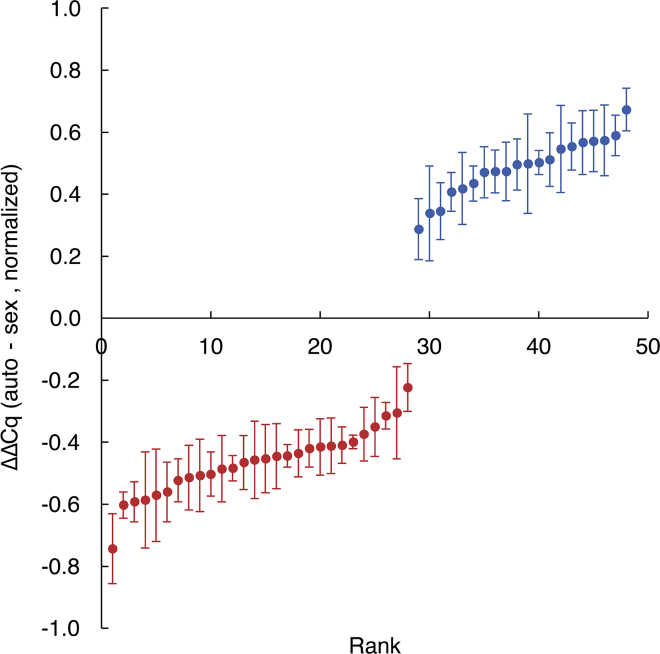
Normalized ΔΔCq values for 28 female (negative values, dark red) and 20 male (positive values, blue) juvenile cuttlefish Error bars represent standard error across 4 technical replicates.

33. For each animal, calculate the Cq difference and the combined standard error:
  ΔCq=A_Cq−S_Cq
  ∑SE=A_SE+S_SE
  a. Normalize ΔCq values to be centered around zero, so that negative values predict females and positive values predict males.
  b. To estimate this offset, use the midpoint value between known males and known females.
  c. Alternatively, you may use the midpoint value between the lowest quartile (females) and highest quartile (males) ΔCq values.
    ΔΔCq=ΔCq−Offset_Cq
    ***Note:*** Ideally Cq values including error bars should fall fully below or fully above zero and should form two clusters, male and female. Single outliers generally indicate a poor DNA sample. If the data do not cluster into two groups, this may indicate insufficient pipetting accuracy when loading the master mix or DNA sample, or a sub-optimal primer set.
34. Assign sex based on ΔΔCq, as in Figure 7:

Female: ΔΔCq < 0.

Male: ΔΔCq > 0.

Assign any individuals whose error bars cross the zero line as low confidence.

## Expected outcomes

Following this protocol enables accurate prediction of sex in groups of cephalopods, such as the 48 dwarf cuttlefish shown here (Figure 7). We previously demonstrated that this approach achieves 100% accuracy, based on post-mortem validation of 81 dwarf cuttlefish.^1^ In our hands, swabbing 48 animals, extracting genomic DNA, performing qPCR, and analyzing the results can all be completed within a single day.

This protocol is broadly applicable across species and sample types. We provide validated primer sequences for seven cephalopod species: *O. bimaculoides, S. officinalis, A. bandense, E. berryi, D. pealeii, I. illecebrosus,* and *I. argentinus* (Table 1). For additional species, we outline a framework for designing and validating qPCR primers that detect the expected two-fold dosage difference between males and females (see “primer design and validation (optional)”). The protocol is compatible with multiple sample types, including non-invasive skin swabs from live animals (“skin swabbing”) and tissue samples such as arm tips or embryos. In all cases, genomic DNA is extracted using the Monarch Spin gDNA Extraction Kit and used as input for qPCR. Finally, this protocol can be used on living animals at a range of life stages, including hatchlings (Figure 2C), juveniles (Figures 2A and 2B), and adults, enabling accurate sex prediction well before sexual maturity and facilitating controlled housing and breeding.

## Limitations

This protocol is highly accurate when performed with validated primers, four technical replicates, and careful pipetting. Confidence in sex assignment is strengthened by analyzing multiple samples and observing clear separation between male and female ΔΔCq values. Accordingly, experiments should include at least four animals with both sexes represented.

This protocol requires access to a qPCR instrument. Laboratories without in-house access may use commercial qPCR services. However, cost and turnaround time may limit feasibility, particularly given the need to maintain animals in isolation following swabbing.

## Troubleshooting

### Problem 1

High variability in Cq values between technical replicates (related to step 31).

### Potential solution

Ensure Cq values fall within the validated linear range of the primers (see “primer validation” section). Verify that pipettes are calibrated and pipetting technique is consistent. Confirm plates are centrifuged and reactions are thoroughly mixed prior to thermocycling. Minimize time at room temperature before qPCR.

### Problem 2

High Cq values (late amplification, related to step 31).

### Potential solution

Confirm samples fall within the linear range of each primer pair (see “primer validation” section). If caused by low DNA concentration, re-swab animals using higher pressure to collect more cells (particularly in adults, which may have a mucus layer), elute DNA in a smaller volume, or concentrate existing samples (e.g. dry them in a speedvac and resuspend in a reduced volume).

### Problem 3

ΔΔCq values do not separate into two clusters (male and female, related to step 33).

### Potential solution

Confirm samples fall within the linear range of each primer pair (see “primer validation” section). Normalize DNA sample concentrations so that the Cq values of all samples are similar (using the conversion that a two-fold dilution corresponds to ∼1 Cq shift). Note that minor deviations from 100% primer efficiency are tolerated when concentrations are similar, but larger differences in concentration can obscure clustering.

### Problem 4

Amplification curves are preceded by a large peak and appear abnormal in slope or form (related to step 31).

### Potential solution

Some gDNA extractions may be contaminated with material from the animal that interferes with the qPCR reaction and is not removed by the purification kit (see Figure S4 for an example amplification curve before and after dilution). This can occur with degraded tissue, buccal samples, or overly concentrated swabs. Dilute samples 10x or more in nuclease-free water and optionally heat at 98°C for 5 min before re-running. If issues persist, re-extract DNA or collect cleaner samples (e.g. gentle mantle swabs).

### Problem 5

Incorrect sex prediction for an individual sample (related to step 34).

### Potential solution

If amplification and melt curves are normal, Cq values fall within the linear range, and technical replicates are tight (SEM < 0.1 cycles), consider biological explanations. These could include: (1) copy number variation at the target locus; (2) discordance between genotype and phenotype (e.g. environmental factors altering sex determination, intersex animals, or behavioral sex mimicry); (3) an alternative sex determination system in the species. To distinguish these possibilities, repeat qPCR using independent primer pairs targeting different autosomal and sex-linked loci.

## Resource availability

### Lead contact

Further information and requests for any resources and reagents should be directed to and will be fulfilled by the lead contact, Tessa G. Montague (tessa.montague@columbia.edu).

### Technical contact

Questions regarding technical specifics of the protocol should be directed to the technical contact, Frederick A. Rubino (fred.rubino@gmail.com).

### Materials availability

This study did not generate new unique reagents.

### Data and code availability

This study did not generate code. Raw data reported in this paper will be shared by the lead contact upon request.

## Acknowledgments

We would like to thank Richard Axel for valuable discussions, funding, and mentorship; Ruth Lehmann for funding and mentorship; Aurora Xu (Axel lab), Gabrielle Winters Bostwick, and Sarah Detmering (Crook lab) for testing the protocol and providing feedback; and Thomas Barlow for photographing the swabbing process. F.A.R. was supported by the 10.13039/100005237Helen Hay Whitney Foundation and is supported by the 10.13039/100019686Whitehead Institute (through Dr. Ruth Lehmann); T.G.M. is supported by the 10.13039/100000011HHMI Hanna H. Gray Fellowship; C.J.G., T.J.M., and T.G.M. are supported by the Howard Hughes Medical Institute (HHMI, through Dr. Richard Axel); and G.C.C., S.T.S., and A.D.K. were funded in part through 10.13039/100000002NIH awards R01HG010774 and R35GM148253.

## Author contributions

Conceptualization, F.A.R., C.J.G., G.C.C., A.D.K., and T.G.M.; methodology, F.A.R., C.J.G., T.J.M., and T.G.M.; investigation, F.A.R., C.J.G., and T.G.M.; formal analysis, F.A.R.; resources, T.J.M. and S.T.S.; writing – original draft, F.A.R., T.J.M., and T.G.M.; writing – review and editing, F.A.R., C.J.G., and T.G.M.; visualization, F.A.R., T.J.M., and T.G.M.; project administration, A.D.K. and T.G.M.; supervision, A.D.K. and T.G.M.

## Declaration of interests

The authors declare no competing interests.

## Declaration of generative AI and AI-assisted technologies in the writing process

During the preparation of this work, the authors used ChatGPT for text editing suggestions to improve the clarity of the manuscript. After using this tool, the authors reviewed and edited the content as needed and take full responsibility for the content of the published article.

## Supplemental information

Document S1. Figures S1–S4

Data S1. Housing tray divider wall laser-cutting template (SVG file), related to “Build a housing tray for temporary individual isolation (optional)” step 3a

Table S1. Amino acid sequences for four autosomal genes and four Z-linked genes from eight cephalopod species, related to “Primer design and validation (optional)”
