# Supplementary material for: Protocol for genotyping cephalopod sex using a skin swab and quantitative PCR
Source: STAR Protoc. 2026 Jul 10;7(3):104693. doi: 10.1016/j.xpro.2026.104693 (PMC13380805; doi:10.1016/j.xpro.2026.104693)
Supplement: Document S1. Figures S1–S4 [file mmc1.pdf]

Protocol for genotyping cephalopod sex using a skin swab and quantitative PCR

Supplementary Figures

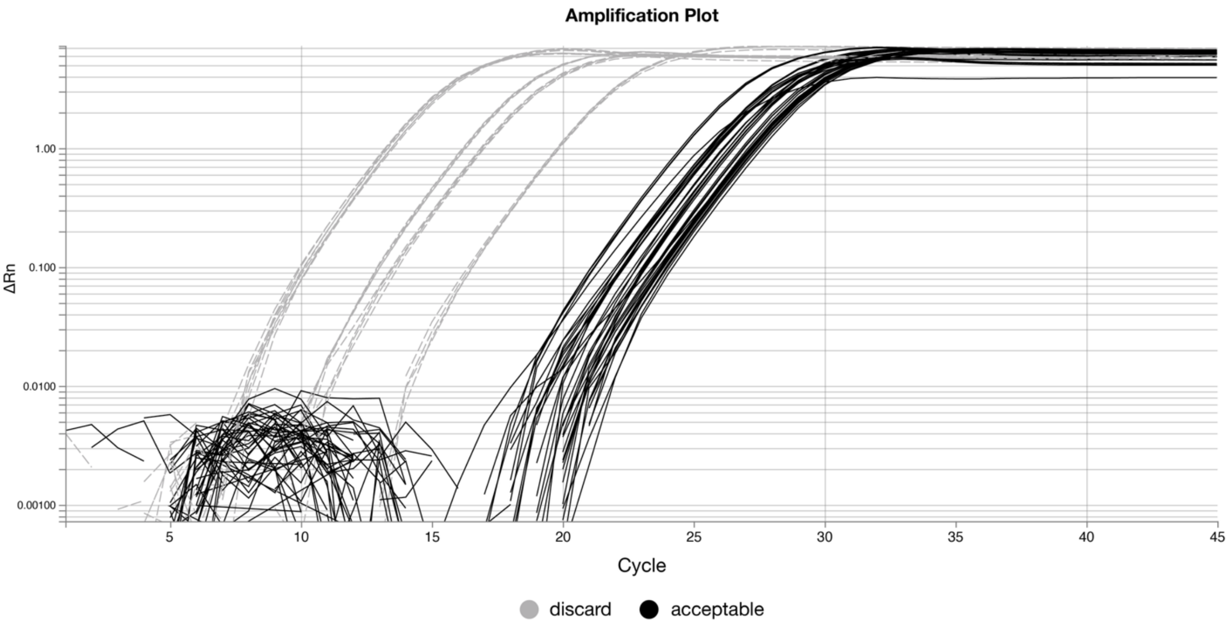

Figure S1. Example of early amplifying primer pairs, related to “**Primer design and validation (optional)**” step 2d-iv. In this example, a group of newly designed primers were tested. Most curves (black) amplified within a small window of Cq values. Some curves (grey, dotted lines) amplified early, indicating off-target amplification and possible amplification of repetitive regions. In examples such as these, black primers should be kept for further analysis while grey primers should be discarded.

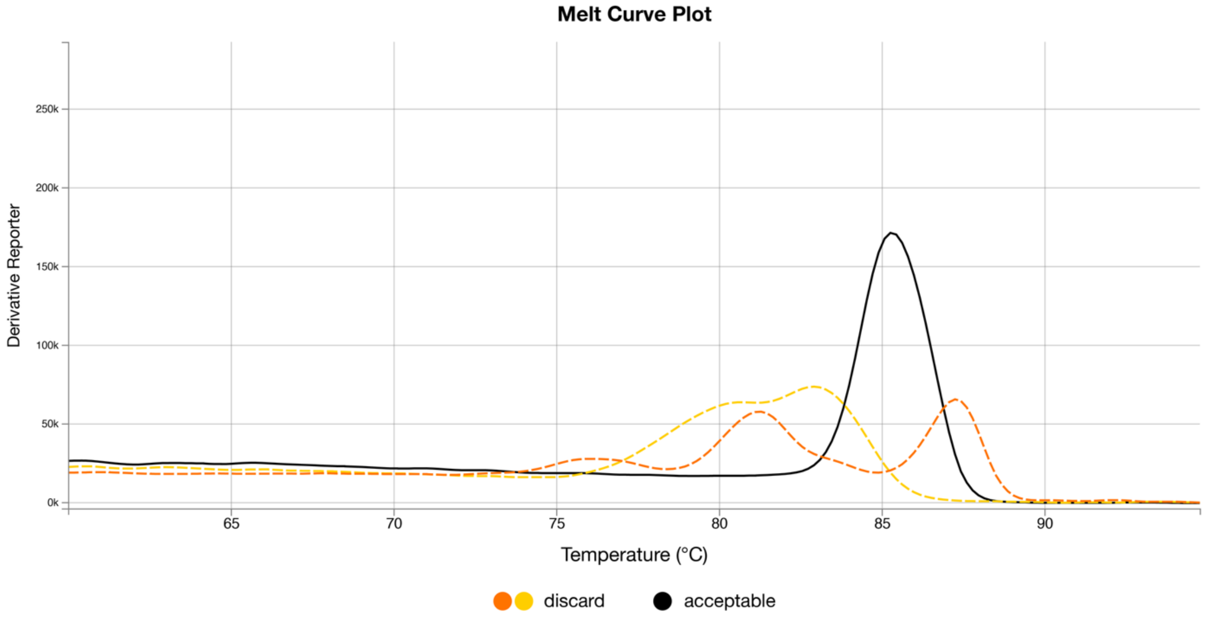

Figure S2. Representative good and poor qPCR melt curves from primer validation, related to “**Primer design and validation (optional)**” step 2e. Orange and yellow dotted traces indicate poorly performing primers that produce multiple amplification products and therefore should be discarded. Black trace shows a well-performing primer pair with a single sharp melting peak consistent with amplification of a single product.

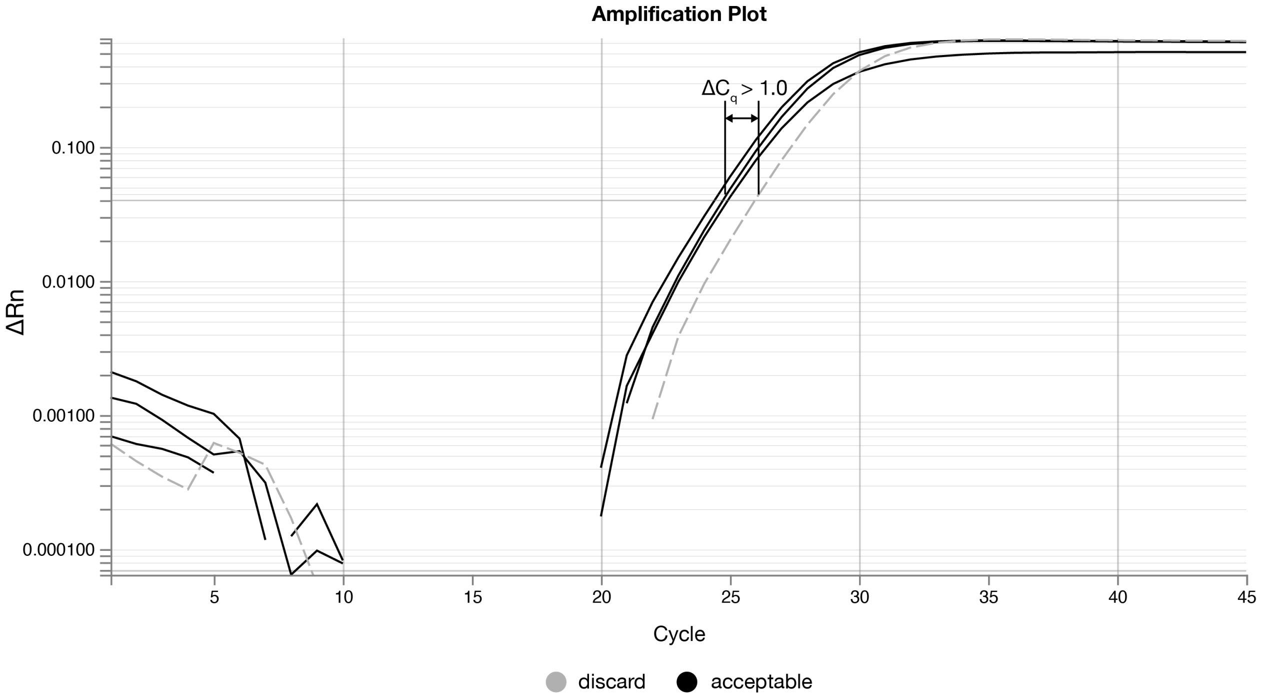

Figure S3. Example of outlier technical replicate removal, related to “**qPCR analysis**” step 31**.** One amplification curve (grey, dotted line) yields a Cq value that differs by more than one cycle from the remaining technical replicates (black lines). Because only a single replicate is affected, the outlier can be removed prior to further analysis.

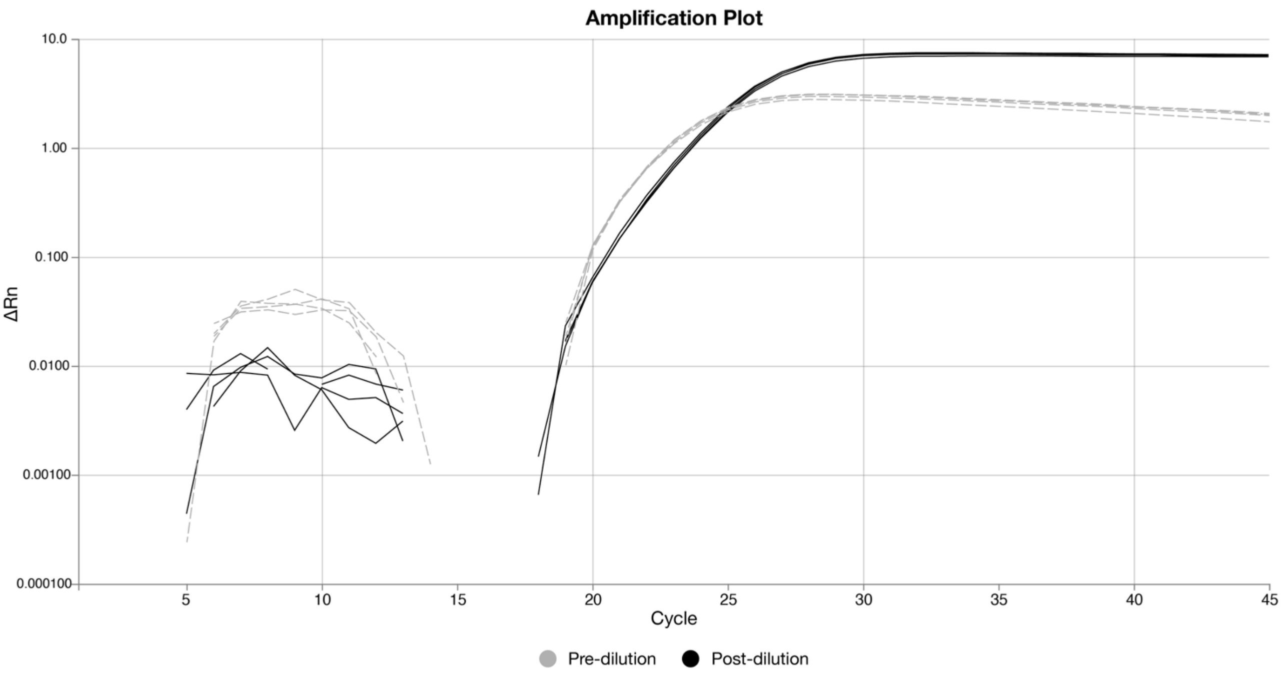

Figure S4. Example amplification curves before and after genomic DNA dilution, related to "**Troubleshooting**" Problem 4**.** The original sample (grey, dotted lines) displayed an early fluorescence hump and an aberrant rounded amplification phase, resulting in rejection during quality control. Following 10× dilution of the genomic DNA sample (black lines), the amplification phase appeared linear on a logarithmic scale, passing quality control.
